# Ethyl 6-chloro-2-methyl-4-phenyl­quinoline-3-carboxyl­ate

**DOI:** 10.1107/S160053680904536X

**Published:** 2009-11-04

**Authors:** R. Subashini, F. Nawaz Khan, Suganya Mittal, Venkatesha R. Hathwar, Seik Weng Ng

**Affiliations:** a Chemistry Division, School of Science and Humanities, VIT University, Vellore 632 014, Tamil Nadu, India; b Solid State and Structural Chemistry Unit, Indian Institute of Science, Bangalore 560 012, Karnataka, India; c Department of Chemistry, University of Malaya, 50603 Kuala Lumpur, Malaysia

## Abstract

In the title compound, C_19_H_16_ClNO_2_, the quinoline ring system is planar (r.m.s. deviation = 0.008 Å). The phenyl group and the –CO_2_ fragment of the ester unit form dihedral angles of 60.0 (1) and 60.5 (1)°, respectively, with the quinoline ring system.

## Related literature

For related structures, see: Baumer *et al.* (2001 ▶); Subashini *et al.* (2009 ▶).
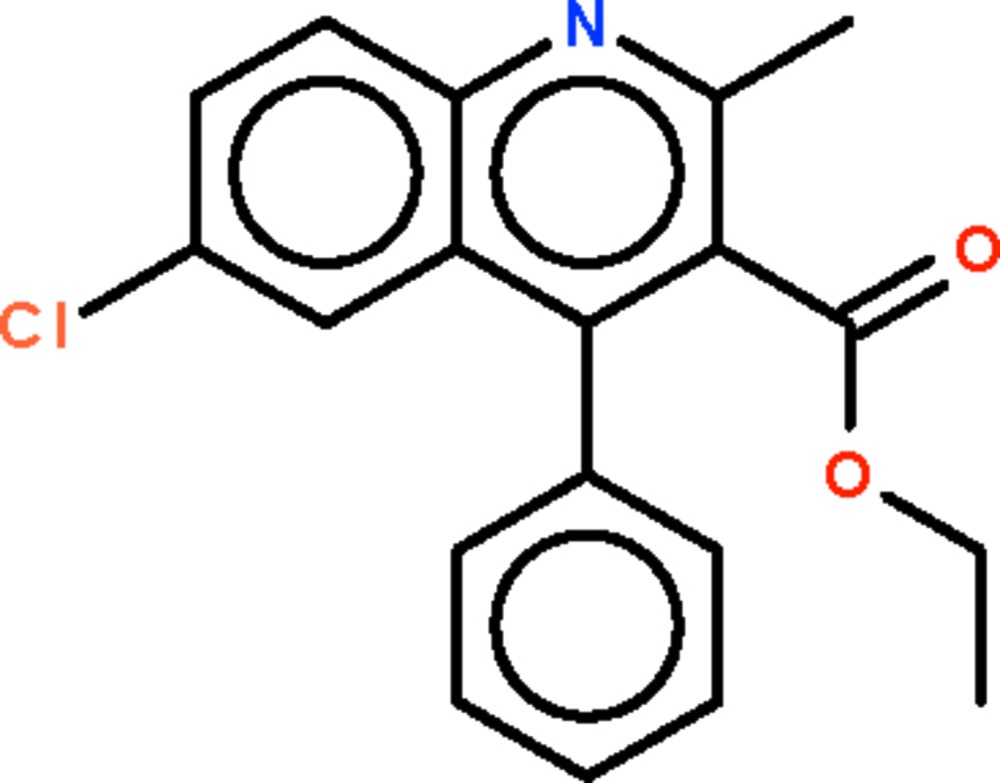


## Experimental

### 

#### Crystal data

C_19_H_16_ClNO_2_
*M* *_r_* = 325.78
Triclinic, 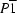

*a* = 8.3622 (3) Å
*b* = 10.1971 (3) Å
*c* = 10.7052 (3) Å
α = 110.440 (2)°
β = 101.588 (2)°
γ = 94.860 (2)°
*V* = 825.91 (4) Å^3^
*Z* = 2
Mo *K*α radiation
μ = 0.24 mm^−1^
*T* = 290 K
0.24 × 0.18 × 0.13 mm

#### Data collection

Bruker SMART CCD area-detector diffractometer
Absorption correction: multi-scan (*SADABS*; Sheldrick, 1996 ▶) *T*_min_ = 0.945, *T*_max_ = 0.970
15008 measured reflections
3775 independent reflections
2854 reflections with *I* > 2σ(*I*)
*R*_int_ = 0.025

#### Refinement

*R*[*F* ^2^ > 2σ(*F* ^2^)] = 0.048
*wR*(*F* ^2^) = 0.147
*S* = 1.04
3775 reflections
210 parameters
1 restraint
H-atom parameters constrained
Δρ_max_ = 0.33 e Å^−3^
Δρ_min_ = −0.38 e Å^−3^

### 

Data collection: *SMART* (Bruker, 2004 ▶); cell refinement: *SAINT* (Bruker, 2004 ▶); data reduction: *SAINT*; program(s) used to solve structure: *SHELXS97* (Sheldrick, 2008 ▶); program(s) used to refine structure: *SHELXL97* (Sheldrick, 2008 ▶); molecular graphics: *X-SEED* (Barbour, 2001 ▶); software used to prepare material for publication: *publCIF* (Westrip, 2009 ▶).

## Supplementary Material

Crystal structure: contains datablocks global, I. DOI: 10.1107/S160053680904536X/ci2957sup1.cif

Structure factors: contains datablocks I. DOI: 10.1107/S160053680904536X/ci2957Isup2.hkl

Additional supplementary materials:  crystallographic information; 3D view; checkCIF report

## Figures and Tables

**Fig. 1.**
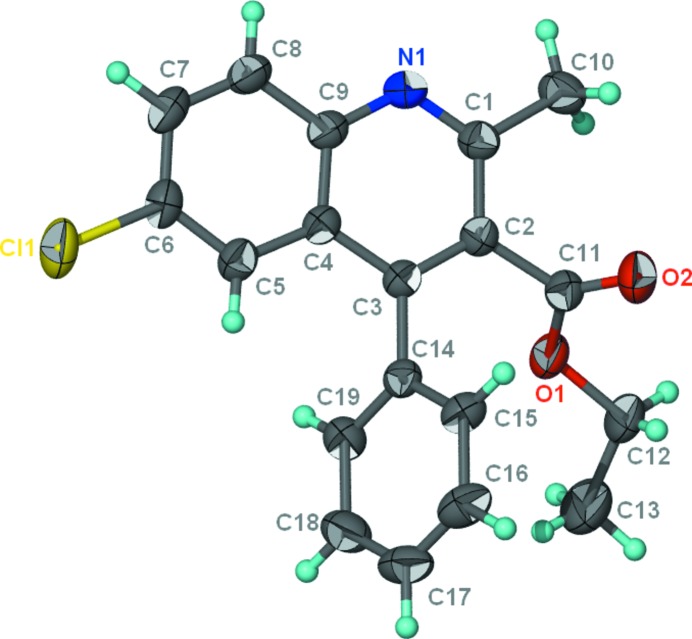
Displacement ellipsoid plot (Barbour, 2001) of C19H16ClNO2 at the 50% probability level. H atoms are drawn as spheres of arbitrary radius.

## supplementary crystallographic information

### Experimental

2-Amino-5-chlorobenzophenone (0.5 mmol), ethyl acetoacete (0.6 mmol, 1.2 equiv.) and iodine (1 mol %) in ethanol (1 ml) were stirred until the reaction was completed, as indicated by TLC. The reaction was quenched with water (15 ml). The organic product was extracted with ethyl acetate. Evaporation of the solvent gave a solid that was recrystallized from DMSO.

### Refinement

C-bound H-atoms were placed in calculated positions (C-H = 0.93–0.97 Å) and were included in the refinement in the riding model approximation, with *U_iso_*(H) set to 1.2*U_eq_*(C). The C—C distance of the ethyl chain was tightly restrained to 1.500 (2) Å.

### Crystal data

**Table:**

C_19_H_16_ClNO_2_	*Z* = 2
*M**_r_* = 325.78	*F*(000) = 340
Triclinic, *P*1	*D*_x_ = 1.310 Mg m^−^^3^
Hall symbol: -P 1	Mo *K*α radiation, λ = 0.71073 Å
*a* = 8.3622 (3) Å	Cell parameters from 1153 reflections
*b* = 10.1971 (3) Å	θ = 1.7–24.3°
*c* = 10.7052 (3) Å	µ = 0.24 mm^−^^1^
α = 110.440 (2)°	*T* = 290 K
β = 101.588 (2)°	Block, colourless
γ = 94.860 (2)°	0.24 × 0.18 × 0.13 mm
*V* = 825.91 (4) Å^3^	

### Data collection

**Table:**

Bruker SMART CCD area-detector diffractometer	3775 independent reflections
Radiation source: fine-focus sealed tube	2854 reflections with *I* > 2σ(*I*)
graphite	*R*_int_ = 0.025
φ and ω scans	θ_max_ = 27.5°, θ_min_ = 2.1°
Absorption correction: multi-scan (*SADABS*; Sheldrick, 1996)	*h* = −10→10
*T*_min_ = 0.945, *T*_max_ = 0.970	*k* = −12→13
15008 measured reflections	*l* = −13→10

### Refinement

**Table:**

Refinement on *F*^2^	Primary atom site location: structure-invariant direct methods
Least-squares matrix: full	Secondary atom site location: difference Fourier map
*R*[*F*^2^ > 2σ(*F*^2^)] = 0.048	Hydrogen site location: inferred from neighbouring sites
*wR*(*F*^2^) = 0.147	H-atom parameters constrained
*S* = 1.04	*w* = 1/[σ^2^(*F*_o_^2^) + (0.0795*P*)^2^ + 0.1903*P*] where *P* = (*F*_o_^2^ + 2*F*_c_^2^)/3
3775 reflections	(Δ/σ)_max_ = 0.001
210 parameters	Δρ_max_ = 0.33 e Å^−^^3^
1 restraint	Δρ_min_ = −0.38 e Å^−^^3^

### Fractional atomic coordinates and isotropic or equivalent isotropic displacement parameters (Å^2^)

**Table:**

	*x*	*y*	*z*	*U*_iso_*/*U*_eq_	
Cl1	0.59565 (8)	0.80991 (7)	0.87661 (5)	0.0711 (2)	
N1	0.79494 (19)	0.38099 (15)	0.42954 (15)	0.0465 (4)	
O1	0.93549 (17)	0.66715 (14)	0.21071 (12)	0.0523 (3)	
O2	0.7449 (2)	0.48238 (16)	0.06011 (14)	0.0690 (4)	
C1	0.8150 (2)	0.40465 (18)	0.31986 (18)	0.0433 (4)	
C2	0.7883 (2)	0.53430 (18)	0.30236 (17)	0.0407 (4)	
C3	0.7375 (2)	0.63972 (17)	0.40021 (16)	0.0387 (4)	
C4	0.71546 (19)	0.61552 (17)	0.52007 (16)	0.0387 (4)	
C5	0.6644 (2)	0.7151 (2)	0.62823 (17)	0.0451 (4)	
H5	0.6412	0.8009	0.6230	0.054*	
C6	0.6496 (2)	0.6845 (2)	0.74006 (17)	0.0481 (4)	
C7	0.6778 (2)	0.5543 (2)	0.74986 (18)	0.0523 (5)	
H7	0.6647	0.5352	0.8264	0.063*	
C8	0.7245 (2)	0.4561 (2)	0.64596 (19)	0.0508 (4)	
H8	0.7428	0.3696	0.6520	0.061*	
C9	0.7459 (2)	0.48359 (18)	0.52871 (17)	0.0422 (4)	
C10	0.8700 (3)	0.2882 (2)	0.2144 (2)	0.0569 (5)	
H10A	0.8926	0.2145	0.2484	0.085*	
H10B	0.9685	0.3259	0.1968	0.085*	
H10C	0.7839	0.2500	0.1307	0.085*	
C11	0.8176 (2)	0.55604 (19)	0.17664 (18)	0.0465 (4)	
C12	0.9625 (4)	0.7067 (2)	0.0980 (2)	0.0733 (7)	
H12A	0.8579	0.6918	0.0321	0.088*	
H12B	1.0348	0.6487	0.0514	0.088*	
C13	1.0409 (5)	0.8617 (3)	0.1562 (3)	0.1064 (11)	
H13A	1.0569	0.8904	0.0826	0.160*	
H13B	1.1459	0.8749	0.2191	0.160*	
H13C	0.9696	0.9181	0.2038	0.160*	
C14	0.7035 (2)	0.77328 (18)	0.38081 (17)	0.0408 (4)	
C15	0.5843 (2)	0.7689 (2)	0.26713 (19)	0.0492 (4)	
H15	0.5239	0.6823	0.2036	0.059*	
C16	0.5557 (3)	0.8940 (2)	0.2488 (2)	0.0609 (5)	
H16	0.4755	0.8908	0.1733	0.073*	
C17	0.6458 (3)	1.0230 (2)	0.3421 (3)	0.0625 (6)	
H17	0.6263	1.1064	0.3293	0.075*	
C18	0.7641 (3)	1.0279 (2)	0.4538 (2)	0.0629 (5)	
H18	0.8251	1.1147	0.5165	0.075*	
C19	0.7927 (2)	0.9034 (2)	0.4729 (2)	0.0521 (4)	
H19	0.8731	0.9075	0.5487	0.063*	

### Atomic displacement parameters (Å^2^)

**Table:**

	*U*^11^	*U*^22^	*U*^33^	*U*^12^	*U*^13^	*U*^23^
Cl1	0.0823 (4)	0.0947 (5)	0.0429 (3)	0.0315 (3)	0.0267 (2)	0.0238 (3)
N1	0.0520 (9)	0.0403 (8)	0.0503 (8)	0.0080 (6)	0.0129 (7)	0.0206 (7)
O1	0.0608 (8)	0.0592 (8)	0.0419 (6)	0.0060 (6)	0.0191 (6)	0.0223 (6)
O2	0.0949 (12)	0.0642 (9)	0.0385 (7)	0.0010 (8)	0.0144 (7)	0.0118 (6)
C1	0.0431 (9)	0.0404 (9)	0.0472 (9)	0.0068 (7)	0.0130 (7)	0.0163 (7)
C2	0.0416 (9)	0.0427 (9)	0.0395 (8)	0.0072 (7)	0.0117 (7)	0.0164 (7)
C3	0.0387 (8)	0.0410 (9)	0.0380 (8)	0.0071 (7)	0.0089 (6)	0.0170 (7)
C4	0.0372 (8)	0.0431 (9)	0.0374 (8)	0.0062 (7)	0.0080 (6)	0.0179 (7)
C5	0.0446 (9)	0.0547 (10)	0.0413 (8)	0.0146 (8)	0.0125 (7)	0.0221 (8)
C6	0.0439 (9)	0.0630 (11)	0.0366 (8)	0.0080 (8)	0.0109 (7)	0.0176 (8)
C7	0.0528 (10)	0.0662 (12)	0.0395 (9)	−0.0035 (9)	0.0069 (7)	0.0274 (9)
C8	0.0581 (11)	0.0494 (10)	0.0470 (9)	0.0005 (8)	0.0069 (8)	0.0260 (8)
C9	0.0418 (9)	0.0440 (9)	0.0422 (8)	0.0033 (7)	0.0077 (7)	0.0201 (7)
C10	0.0645 (12)	0.0455 (10)	0.0657 (12)	0.0169 (9)	0.0276 (10)	0.0186 (9)
C11	0.0557 (10)	0.0464 (10)	0.0422 (9)	0.0155 (8)	0.0176 (8)	0.0177 (8)
C12	0.113 (2)	0.0663 (14)	0.0491 (11)	0.0044 (13)	0.0366 (12)	0.0246 (10)
C13	0.165 (3)	0.086 (2)	0.0744 (17)	−0.011 (2)	0.0446 (19)	0.0358 (15)
C14	0.0453 (9)	0.0429 (9)	0.0410 (8)	0.0124 (7)	0.0171 (7)	0.0192 (7)
C15	0.0503 (10)	0.0527 (10)	0.0515 (10)	0.0093 (8)	0.0119 (8)	0.0280 (8)
C16	0.0559 (11)	0.0731 (14)	0.0729 (13)	0.0208 (10)	0.0185 (10)	0.0469 (12)
C17	0.0709 (13)	0.0522 (12)	0.0866 (15)	0.0251 (10)	0.0355 (12)	0.0407 (11)
C18	0.0757 (14)	0.0423 (10)	0.0725 (13)	0.0136 (10)	0.0271 (11)	0.0180 (10)
C19	0.0583 (11)	0.0474 (10)	0.0488 (10)	0.0107 (8)	0.0128 (8)	0.0158 (8)

### Geometric parameters (Å, °)

**Table:**

Cl1—C6	1.7414 (19)	C10—H10A	0.96
N1—C1	1.317 (2)	C10—H10B	0.96
N1—C9	1.364 (2)	C10—H10C	0.96
O1—C11	1.329 (2)	C12—C13	1.513 (2)
O1—C12	1.450 (2)	C12—H12A	0.97
O2—C11	1.206 (2)	C12—H12B	0.97
C1—C2	1.428 (2)	C13—H13A	0.96
C1—C10	1.503 (3)	C13—H13B	0.96
C2—C3	1.380 (2)	C13—H13C	0.96
C2—C11	1.501 (2)	C14—C19	1.383 (3)
C3—C4	1.432 (2)	C14—C15	1.394 (2)
C3—C14	1.489 (2)	C15—C16	1.389 (3)
C4—C5	1.416 (2)	C15—H15	0.93
C4—C9	1.421 (2)	C16—C17	1.381 (3)
C5—C6	1.364 (2)	C16—H16	0.93
C5—H5	0.93	C17—C18	1.373 (3)
C6—C7	1.404 (3)	C17—H17	0.93
C7—C8	1.362 (3)	C18—C19	1.387 (3)
C7—H7	0.93	C18—H18	0.93
C8—C9	1.419 (2)	C19—H19	0.93
C8—H8	0.93		
			
C1—N1—C9	118.67 (14)	H10B—C10—H10C	109.5
C11—O1—C12	115.60 (15)	O2—C11—O1	124.32 (17)
N1—C1—C2	122.09 (16)	O2—C11—C2	124.75 (18)
N1—C1—C10	116.33 (15)	O1—C11—C2	110.93 (14)
C2—C1—C10	121.57 (15)	O1—C12—C13	108.36 (17)
C3—C2—C1	120.68 (15)	O1—C12—H12A	110.0
C3—C2—C11	119.90 (15)	C13—C12—H12A	110.0
C1—C2—C11	119.42 (15)	O1—C12—H12B	110.0
C2—C3—C4	117.83 (14)	C13—C12—H12B	110.0
C2—C3—C14	121.20 (14)	H12A—C12—H12B	108.4
C4—C3—C14	120.96 (14)	C12—C13—H13A	109.5
C5—C4—C9	119.10 (15)	C12—C13—H13B	109.5
C5—C4—C3	123.49 (15)	H13A—C13—H13B	109.5
C9—C4—C3	117.41 (15)	C12—C13—H13C	109.5
C6—C5—C4	119.64 (16)	H13A—C13—H13C	109.5
C6—C5—H5	120.2	H13B—C13—H13C	109.5
C4—C5—H5	120.2	C19—C14—C15	118.78 (16)
C5—C6—C7	122.02 (17)	C19—C14—C3	120.68 (15)
C5—C6—Cl1	119.74 (15)	C15—C14—C3	120.52 (16)
C7—C6—Cl1	118.23 (13)	C16—C15—C14	120.00 (18)
C8—C7—C6	119.22 (16)	C16—C15—H15	120.0
C8—C7—H7	120.4	C14—C15—H15	120.0
C6—C7—H7	120.4	C17—C16—C15	120.37 (19)
C7—C8—C9	121.19 (17)	C17—C16—H16	119.8
C7—C8—H8	119.4	C15—C16—H16	119.8
C9—C8—H8	119.4	C18—C17—C16	119.94 (18)
N1—C9—C8	117.90 (15)	C18—C17—H17	120.0
N1—C9—C4	123.31 (15)	C16—C17—H17	120.0
C8—C9—C4	118.79 (16)	C17—C18—C19	119.9 (2)
C1—C10—H10A	109.5	C17—C18—H18	120.0
C1—C10—H10B	109.5	C19—C18—H18	120.0
H10A—C10—H10B	109.5	C14—C19—C18	120.97 (19)
C1—C10—H10C	109.5	C14—C19—H19	119.5
H10A—C10—H10C	109.5	C18—C19—H19	119.5
			
C9—N1—C1—C2	0.5 (3)	C7—C8—C9—C4	−0.8 (3)
C9—N1—C1—C10	179.69 (16)	C5—C4—C9—N1	179.81 (15)
N1—C1—C2—C3	−1.0 (3)	C3—C4—C9—N1	0.2 (2)
C10—C1—C2—C3	179.86 (16)	C5—C4—C9—C8	−0.2 (2)
N1—C1—C2—C11	178.43 (16)	C3—C4—C9—C8	−179.76 (15)
C10—C1—C2—C11	−0.7 (3)	C12—O1—C11—O2	6.4 (3)
C1—C2—C3—C4	1.1 (2)	C12—O1—C11—C2	−174.03 (17)
C11—C2—C3—C4	−178.39 (15)	C3—C2—C11—O2	−119.9 (2)
C1—C2—C3—C14	−177.78 (15)	C1—C2—C11—O2	60.7 (3)
C11—C2—C3—C14	2.8 (2)	C3—C2—C11—O1	60.5 (2)
C2—C3—C4—C5	179.76 (15)	C1—C2—C11—O1	−118.93 (17)
C14—C3—C4—C5	−1.4 (2)	C11—O1—C12—C13	156.4 (2)
C2—C3—C4—C9	−0.7 (2)	C2—C3—C14—C19	−119.39 (19)
C14—C3—C4—C9	178.17 (15)	C4—C3—C14—C19	61.8 (2)
C9—C4—C5—C6	1.7 (3)	C2—C3—C14—C15	58.7 (2)
C3—C4—C5—C6	−178.74 (16)	C4—C3—C14—C15	−120.12 (18)
C4—C5—C6—C7	−2.3 (3)	C19—C14—C15—C16	−0.7 (3)
C4—C5—C6—Cl1	177.19 (13)	C3—C14—C15—C16	−178.83 (17)
C5—C6—C7—C8	1.3 (3)	C14—C15—C16—C17	0.5 (3)
Cl1—C6—C7—C8	−178.20 (14)	C15—C16—C17—C18	−0.1 (3)
C6—C7—C8—C9	0.3 (3)	C16—C17—C18—C19	−0.2 (3)
C1—N1—C9—C8	179.84 (16)	C15—C14—C19—C18	0.5 (3)
C1—N1—C9—C4	−0.1 (3)	C3—C14—C19—C18	178.57 (18)
C7—C8—C9—N1	179.20 (17)	C17—C18—C19—C14	0.0 (3)
